# Rational mpox vaccine design: immunogenicity and protective effect of individual and multicomponent proteins in mice

**DOI:** 10.1080/22221751.2025.2482702

**Published:** 2025-03-19

**Authors:** Xueting Cheng, Yawei Wang, Baoying Huang, Jialuo Bing, Tangqi Wang, Ruiwen Han, Shuting Huo, Shucai Sun, Li Zhao, Chang Shu, Yao Deng, Wenjie Tan

**Affiliations:** National Key Laboratory of Intelligent Tracking and Forecasting for Infectious Diseases, Key Laboratory of Biosafety, National Health Commissions, National Institute for Viral Disease Control and Prevention, China CDC, Beijing, People’s Republic of China

**Keywords:** Orthopoxvirus, mpox virus, multicomponent protein vaccines, immunogenicity, cross-protection

## Abstract

The 2022 global mpox virus (MPXV) outbreak highlights the urgent need for safer, next-generation vaccines. We compared the immunogenicity and protective efficacy of individual and multicomponent membrane proteins of MPXV virions in mice to inform the development of a recombinant subunit vaccine against mpox. BALB/c mice were immunized with eukaryotically expressed A35R, A29L, B6R, and M1R proteins, administered individually or in multicomponent combinations with an Al(OH)_3_ + CpG oligodeoxynucleotide adjuvant. Three multicomponent protein vaccines (A29/B6, A29/B6/M1, and A29/B6/M1/A35) provided complete protection, but others (individual protein and A35/M1 combinations) provided partial protection against challenge with high-lethal doses of vaccinia virus Western Reserve (VACV-WR). Additionally, A29/B6 conferred partial protection, whereas A29/B6/M1 and A29/B6/M1/A35 provided complete protection against ectromelia virus (ECTV), with A29/B6/M1 being most effective. All vaccines induced strong antigen-specific immunoglobulin G (IgG) and cellular immunity, whereas only four (M1, A35/M1, A29/B6/M1, A29/B6/M1/A35) exhibited significant neutralizing activity against MPXV, VACV-Tiantan, and ECTV. Correlation analysis suggested that neutralizing antibodies and A35-/A29-/B6-specific cellular immunity act as complementary defense mechanisms, potentially providing first- and second-line protection against MPXV and related orthopoxviruses. Collectively, A29/B6/M1 demonstrated the best protective efficacy. This study provides novel insights into immunogen optimization and potential mechanisms for the development of vaccines against MPXV and other orthopoxviruses.

## Introduction

Mpox is a zoonotic disease caused by the mpox virus (MPXV) [1]. Since November 2022, the outbreak of mpox has led to more than 117,663 infections in 127 countries worldwide, many in which mpox was not previously considered endemic. On August 14, 2024, the World Health Organization declared mpox a Public Health Emergency of International Concern for the second time in 2 years [2]. Currently approved MPXV vaccines are based primarily on live, attenuated smallpox vaccines such as modified vaccinia Ankara-Bavarian Nordic (MVA-BN) [3]. Although previous studies suggest that smallpox vaccines confer some cross-protection against MPXV, including emerging strains, the safety of attenuated smallpox vaccines has raised concern regarding their use [3–6]. ACAM2000, a second-generation live attenuated vaccinia virus vaccine, is associated with a risk of cardiac complications, including myocarditis, which is reported in approximately one in 20,000 vaccinated individuals [7–9]. MVA-BN (JYNNEOS), a third-generation, non-replicating live attenuated MVA vaccine, has been approved in multiple countries for the prevention of both smallpox and mpox [10]. MVA-BN appears to offer a safer alternative to live attenuated vaccinia vaccines; the U.S. Food and Drug Administration recommends intradermal administration of one-fifth of the standard subcutaneous dose for at-risk individuals over 18 years of age. Although this dose-sparing strategy increases vaccine availability, it results in reduced MPXV neutralizing antibody levels [8,11]. Additionally, challenges such as vaccine availability, rapid manufacturing requirements, and the extensive animal reservoir of MPXV highlights the urgent need for a safer and more accessible vaccines capable of effectively controlling mpox and potentially preventing diseases caused by other orthopoxviruses, particularly as MPXV continues to evolve and the risk of human-to-human transmission increases [12].

MPXV is an enveloped, double-stranded DNA virus that belongs to the Poxviridae family, along with the variola virus (VARV), vaccinia virus (VACV), and ectromelia virus (ECTV) [13]. Its genome is approximately 197 kb in length and encodes approximately 190 proteins, many of which are highly conserved across Orthopoxvirus species [14]. Therefore, infection with, or immunity to, one species of Orthopoxvirus may confer cross-protection against infection with other orthopoxviruses [15]. Several approved vaccines are available for the immunization and post-exposure prophylaxis of individuals at high risk of exposure to emergent orthopoxviruses [16].

Intracellular mature virions (IMV) and extracellular enveloped virions (EEV) are two key forms of the virus involved in its spread between host cells [17], each possessing a distinct set of surface antigens that serve as immunological targets [14]. In this study, two EEV antigens (A35R and B6R) and two IMV antigens (A29L and M1R) were selected based on their critical structural roles and well-characterized immunogenic properties against VARV, VACV, and MPXV, demonstrated in previous studies [18–20]. These antigen combinations have been extensively studied using multiple vaccine platforms and have been shown to exhibit high immunogenicity and confer protection against lethal Orthopoxvirus infections in both mice and rhesus macaques [21–27]. Specifically, A29L and M1R are key IMV surface proteins involved in viral entry and serve as potent targets for neutralizing antibodies [28,29], whereas B6R, an EEV glycoprotein, facilitates viral dissemination and plays a crucial role in immune recognition [30,31]. Additionally, A35R contributes to virion cell-to-cell spread and modulates complement-driven cytolysis [32]. These four antigens offer a well-balanced selection targeting both IMV and EEV forms, ensuring broad immune coverage thus enhancing the vaccine's protective potential. Furthermore, these antigens are highly conserved among three orthopoxviruses that are highly pathogenic to humans (Table S1), suggesting the potential for cross-protection. Immune responses to these antigens persist for decades following VACV immunization or recovery from MPXV infection, highlighting their importance in conveying long-term immunity [33,34].

Recently, multiple vaccine platforms using IMV- and EEV-derived multivalent MPXV antigens (A29L, A35R, B6R, and M1R) have been widely studied. Previous studies indicate their potential to trigger strong immune responses and protect mice and macaques against severe Orthopoxvirus infections [35–42]. Protein vaccines have good safety profiles, and combination with adjuvants enhances their protective effect [25,42,43], making them the best option for immunocompromised individuals. Recent studies have shown that intradermal inoculation with three doses of a mixture of A29L, M1R, A35R, and B6R proteins linked to the QS-21 adjuvant activates both cellular and humoral immunity, inducing high titers of binding and neutralizing antibodies that inhibit viral replication and protect mice from MPXV infection [42]. However, the immunogenicity, protective effects, and the immune correlates of these antigens remain unclear.

Therefore, in this study, we focused on subunit vaccines, aiming to optimize the immunogen and understand potential mechanisms for developing novel vaccines against MPXV and other orthopoxviruses.

## Materials and methods

### Ethics statement

All procedures involving mice were approved by the Animal Testing Committee of the Chinese Center for Disease Control and Prevention (approval number: bdbs20240703047). The experiments were carried out in accordance with the *Chinese Laboratory Animal Regulations* and the guidelines on *Laboratory Animal Environment and Housing Facilities Requirements*.

### Cells and viruses

HEK293T, chicken embryo fibroblast (CEF), and Vero cells were cultured in Dulbecco’s modified Eagle’s medium (Gibco, USA) supplemented with 10% fetal bovine serum (FBS; Gibco) and 1% penicillin–streptomycin (PS; Gibco) in a 5% CO_2_ incubator at 37°C. The MPXV (clade IIb, B.1.3) were propagated and titrated in Vero cells under Biosafety Level 3 conditions. The VACV-WR strain and ECTV were propagated in CEF cells and titrated in Vero cells for mouse challenge. The Tiantan strain of vaccinia virus (VTT) and ECTV carrying fluorescent genes were constructed in our laboratory for neutralization assays.

### Protein expression and purification

MPXV (accession number AF380138.1) recombinant proteins of A29L, M1R, A35R, and B6R were inserted into the pVRC vector and marked with a His-tag respectively, as described previously [44]. Each of these sequences was separately tagged with a His-tag to facilitate protein purification. Subsequently, the plasmids were transiently transfected into HEK293T cells, and the supernatant was then collected. Protein purification was performed using immobilized metal affinity chromatography (HIS Select, Cytiva). Elution was carried out using 50- and 300-mM imidazole. The imidazole was then removed through an ultrafiltration centrifuge tube (Merck Millipore), and the protein was collected in phosphate-buffered saline (PBS). Protein concentration and purity were assessed using western blot and Coomassie blue staining, respectively. Finally, proteins with a purity greater than 90% were quantified using the bicinchoninic acid assay.

### Vaccination and mouse challenge experiment

Specific-pathogen-free (SPF) 6–8-week-old female BALB/c mice were randomly divided into groups. Each group received a mixture of 10 µg each of the protein with 100 µg of alum (Sinovac Biotech Co., Beijing, China) and 10 µg of CpG ODN 1826 (Synbio Technologies, Suzhou, China) via the intramuscular (IM) route.

Mice that received a mixture of Al(OH)3 + CpG ODN 1826 in physiological saline were used as a negative control. Immunizations were performed on Days 0 and 28. On Day 49 post-immunization, the mice were intranasally challenged with 15 LD_50_ of VACV-WR (6 × 10^6^ PFU) and 15 LD_50_ of ECTV (120 PFU). Changes in body weight and survival rates were monitored over 14 days. Some infected mice were euthanized 7 days post-challenge for tissue collection and virological analysis.

### ELISA

Enzyme-linked immunosorbent assay (ELISA) plates (Corning) were coated with 100 ng of specific viral proteins (A29L, B6R, M1R, and A35R) overnight at 4°C. The plates were then blocked with PBS containing 10% FBS before adding diluted serum samples. After washing, an horseradish peroxidase (HRP)-conjugated goat anti-mouse IgG antibody (ZSGB-Bio, Beijing, China) diluted in 2% FBS in PBS was added and incubated at 37°C for 1 h. Colorimetric detection was performed by adding 100 µL of 3,3',5,5'-tetramethylbenzidine (TMB) substrate (Solarbio, Beijing, China) per well and incubating at room temperature for 2 min. The reaction was stopped with 1M H_2_SO_4_, and absorbance was measured at 450 nm. Endpoint titers were defined as the serum dilution producing an absorbance value at least 2.1-fold greater than that of the mean of the negative control.

### Neutralization assay

The MPXV strain Claret IIb, B.1.3, was maintained in our laboratory [45]. Plaque reduction neutralization tests (PRNT) for MPXV were performed as previously described [46]. Briefly, Vero cells were seeded in 24-well plates and cultured overnight. Approximately 100 PFU of the virus was mixed with serially diluted serum (starting at 1:20) and added to the cells. After a 96-h incubation period, the samples were plaque titrated, stained with crystal violet, and the number of plaques was enumerated. Neutralizing antibody titers were defined as the highest serum dilution that resulted in a 50% reduction (NT_50_) in the number of virus plaques. All experiments involving MPXV were conducted in a Biosafety Level 3 laboratory.

Neutralization assays based on luciferase reporter systems (VTT-Luc and ECTV-Gluc) were performed to measure fluorescent reporter gene expression, according to previously described methods [47]. Serial diluted samples (starting at 1:30) were mixed in equal volumes with 200 PFU of VTT-Luc or 100 PFU of ECTV-Gluc and incubated at 37°C with 5% CO_2_ for 1 h. Subsequently, Vero cells (4 × 10⁴ cells/well) were added to each well and incubated for 48 h. Luciferase activity was measured using the Bright-Glo Luciferase Assay System (Vazyme, Nanjing, China). The NT_50_ for each serum sample was defined as the dilution at which luminescence decreased by 50% relative to that in virus-containing control wells, after background subtraction from cell-only control wells.

### ELISpot assay

Cellular immune responses in mice vaccinated with the subunit vaccine were assessed using interferon gamma (IFN-γ) ELISpot kits (BioLegend, San Diego, CA, USA) according to the manufacturer’s instructions. Immunized mouse splenocytes (5 × 10⁵ cells/well) were stimulated with MPXV A29L and B6R peptide pools and A35R and M1R proteins. After 36 h of incubation at 37°C in 5% CO_2_, plates were washed and treated sequentially with biotinylated anti-mouse IFN-γ antibody for 2 h and streptavidin-HRP for 1 h, both at room temperature. TMB substrate was added to visualize spots, which were then scanned and quantified using an ImmunoSpot CTL reader (Cellular Technology Limited, Cleveland, OH, USA). Spot-forming units per million cells were calculated by subtracting the values of the negative control.

### Evaluation of protection in mice after VACV-WR and ECTV challenge

Seven days after the VACV-WR challenge, the mice were euthanized, and lung tissues were harvested (four mice per group). The liver, spleen, lung, and whole blood were collected from challenged mice 7 days after the ECTV challenge. Tissue homogenates were clarified via centrifugation at 1000×*g* for 10 min. Infectious virus particles in the supernatants were quantified on Vero cells using the 50% tissue culture infectious dose (TCID_50_). Viral nucleic acids were extracted using Viral DNA Extraction Kits (Vazyme, Nanjing, China) according to the manufacturer's instructions. DNA quantification was performed using quantitative polymerase chain reaction (qPCR) with Probe qPCR Mix (Vazyme), with qPCR conducted on a LightCycler 96 Instrument (Roche Diagnostics Ltd., Rotkreutz, Switzerland) [47].

Necropsies were conducted on four mice per group Day 7 post infection in accordance with standard procedures. Lung tissues were fixed in 4% neutral buffered formaldehyde, embedded in paraffin, and stained with hematoxylin and eosin. The stained sections were examined by a veterinary pathologist, who evaluated the severity of lesions using a standardized four-tier scoring system to facilitate comparisons across groups.

### Statistical analysis

Data were analyzed using GraphPad Prism 9 software (GraphPad Software, San Diego, CA, USA). The significance of differences between groups was assessed using independent samples t-tests for comparisons of two groups, and analysis of variance (ANOVA) for comparisons of multiple groups. Correlations were assessed using Spearman correlation coefficients. All hypothesis tests were conducted using a two-tailed approach, and *p* values <0.05 were considered statistically significant. Data were presented as the mean ± SEM.

## Results

### Characterization of the effects of recombinant MPXV protein subunit vaccines in mice

**Figure 1.**
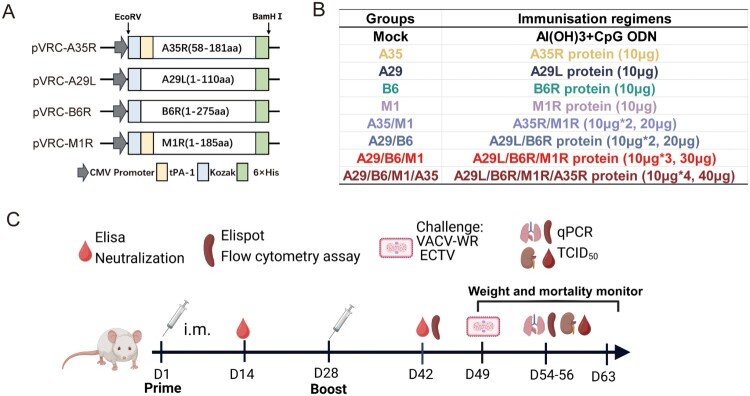
Purification and characterization of MPXV A35R, A29L, B6R, M1R protein and immune program. (A) Construction of eukaryotic vectors expressing MPXV A35R, A29L, B6R, and M1R. (B) Animal experiment grouping. (C) Schematic diagram of immunization and challenge experiment.

EcoRV and BamHI restriction sites, signal peptide sequences, and a 6×His-tag were included to facilitate subsequent protein purification (Figure 1(A)). Western blotting confirmed the expression of MPXV proteins A35R, A29L, B6R, and M1R (Figure S1A). Coomassie brilliant blue staining and sodium dodecyl-sulfate polyacrylamide gel electrophoresis confirmed that the purity of the A35R, A29L, B6R, and M1R proteins exceeded 90% (Figure S1B).

To develop a recombinant subunit vaccine against mpox, we compared the immunogenicity and protective efficacy induced by individual or multicomponent membrane proteins from virions of MPXV in mice. BALB/c mice were inoculated with subunit vaccines derived from eukaryotic expressed individually protein (A35R, A29L, B6R, and M1R) or in multicomponent combinations with an Al (OH)_3_ + CpG ODN adjuvant (Figure 1(C)).

### Three multicomponent protein vaccines provided complete protection, but others provided partial protection against a challenge with a high-lethal dose of VACV-WR

**Figure 2.**
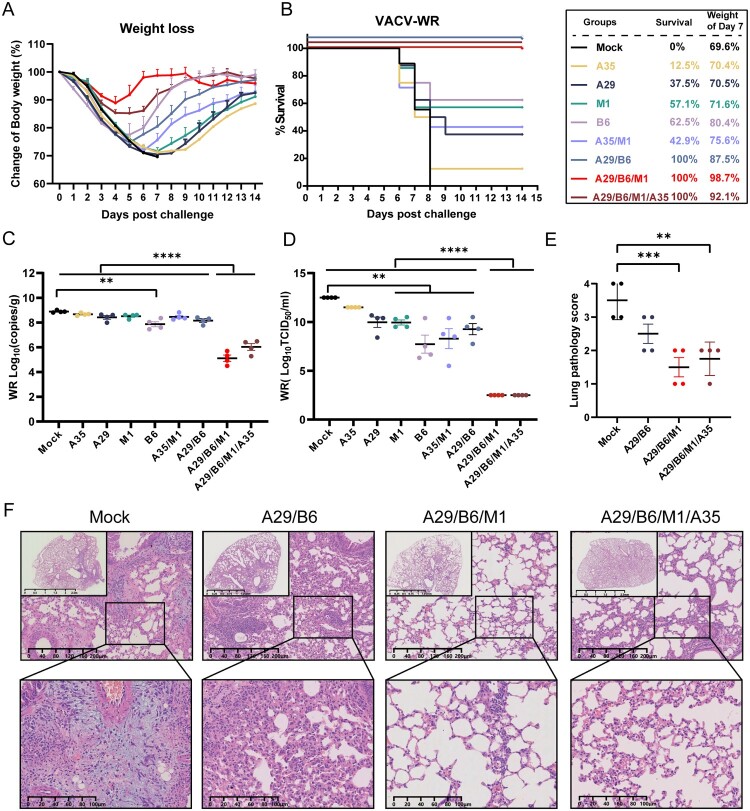
Cross-protection by multivalent MPXV subunit vaccines against lethal VACV-WR challenge in mice. (A,B) Changes in body weight and survival after intranasal challenge with 15 LD_50_ of VACV-WR on Day 7 after administering the second dose of vaccine evaluated in mice vaccinated with subunit vaccines. (C) Virus copies numbers and (D) virus titers in lung tissue 7 days post infection. (E) Lung pathological examination and representative images of mice vaccinated with monovalent, multivalent subunit, and mock vaccines (*n* = 4). Data are expressed as the mean ± SEM and were analyzed using one-way ANOVA, with *p* < 0.05 indicating statistical significance.

To evaluate the protective efficacy of MPXV proteins (A35R, A29L, B6R, and M1R), we selected the VACV-WR strain as a substitute strain for MPXV for challenge protection in BABL/c mice. BALB/c mice immunized with individual, bivalent, trivalent, or quadrivalent protein vaccines were challenged with a high-lethal dose of 6 × 10^6^ PFU (15LD_50_) of VACV-WR, administered intranasally. Three multicomponent protein vaccines (A29/B6, A29/B6/M1, and A29/B6/M1/A35), including EEV and IMV, provided complete protection (Figure 2(A,B)). Mice in the A29/B6/M1 group experienced only mild, transient weight loss, and their weight returned to baseline by 7 days post infection (DPI). However, the other vaccines (individual proteins and the A35/M1 combination) provided only partial protection against a challenge with a high-lethal dose of VACV-WR. Among the individual protein vaccines, the B6 vaccine showed the best protection with a survival rate of 62.5%. In contrast, non-immunized mice exhibited continuous weight loss, resulting in death within 6–8 days (Figure 2(A,B)).

To further assess the protective efficacy of the various subunit vaccines, we measured viral titers and viral loads in the lungs of infected mice using qPCR and TCID_50_ assays (Figure 2(C,D)). Owing to the high challenge dose of intranasal VACV-WR, elevated viral loads were detected in the lungs of most groups other than the B6, A29/B6/M1, and A29/B6/M1/A35 groups at 7 DPI. Additionally, compared with the control group, the M1, B6, and multicomponent subunit vaccines effectively inhibited viral replication, with a marked reduction of the viral titers in the lungs of mice in the A29/B6/M1 and A29/B6/M1/A35 groups.

Furthermore, pathological examination of lung tissue at 7 DPI revealed mild pathological changes in the A29/B6/M1 and A29/B6/M1/A35 groups (Figure 2(E,F)), including pulmonary vasodilation, congestion, minimal interstitial inflammatory cell infiltration, and minor alveolar wall thickening. In contrast, the control group displayed moderate to severe interstitial pneumonia, characterized by extensive alveolar cell necrosis, significant congestion, and pronounced inflammatory cell infiltration.

Three multicomponent protein vaccine formulations combining EEV and IMV antigens (A29/B6, A29/B6/M1, and A29/B6/M1/A35) provided complete protection against challenge with a high-lethal dose of VACV-WR, but the other vaccine formulations (individual proteins and A35/M1 combinations) provided only partial protection. The A29/B6/M1 and A29/B6/M1/A35 multicomponent vaccines substantially reduced VACV-WR proliferation in mice, reduced pathological changes in the lungs, and conferred effective protection against infection with a high-lethal dose of VACV-WR.

### Two multicomponent vaccines provided complete cross-protection against a high-lethal dose of ECTV

To assess the cross-protective efficacy of MPXV protein vaccines against orthopoxviruses, multicomponent MPXV protein vaccines A29/B6, A29/B6/M1, and A29/B6/M1/A35 were administered to mice, and the ice were then challenged with a high-lethal dose of ECTV (15 LD_50_, 120 PFU). The A29/B6/M1 and A29/B6/M1/A35 vaccines provided complete protection against ECTV, whereas the A29/B6 vaccine provided partial protection, with a survival rate of 62.5% (Figure 3(A,B)). Unlike the response to the VACV-WR challenge, mice in the control group maintained their body weight for 4 DPI following an intranasal ECTV challenge, then experienced rapid weight loss, leading to death at 6–8 DPI. In contrast, mice in the A29/B6/M1 and A29/B6/M1/A35 groups showed stable body weight until 6 DPI, with minor weight loss between 6 and 11 DPI, followed by a gradual recovery. As ECTV induces systemic infection, high titers of the virus were detected in the lungs, liver, spleen, and blood of the control mice. However, vaccination with multicomponent subunit vaccines (A29/B6/M1 and A29/B6/M1/A35) effectively inhibited viral replication, significantly reducing viral titers in the lungs, liver, spleen, and whole blood. The A29/B6/M1 and A29/B6/M1/A35 vaccines exhibited superior protective efficacy to that of the A29/B6 vaccine (Figure 3(C,D)). Figure 3.Cross-protection by multivalent MPXV subunit protein vaccines against lethal ECTV challenge in mice (A,B) Changes in body weight and survival after intranasal challenge with 15 LD_50_ of ECTV on Day 7 after administering the second dose of vaccine evaluated in mice vaccinated with subunit vaccines. (C) Virus copies numbers and (D) virus titers in the lung, liver, spleen, and blood 7 days post infection. (E) Pathological score and (F) representative images of pathological examination of mice vaccinated with monovalent, multivalent subunit, and mock vaccines (*n* = 4). Data are expressed as the mean ± SEM and were analyzed using one-way ANOVA, with *p* < 0.05 indicating statistical significance.

**Figure:**
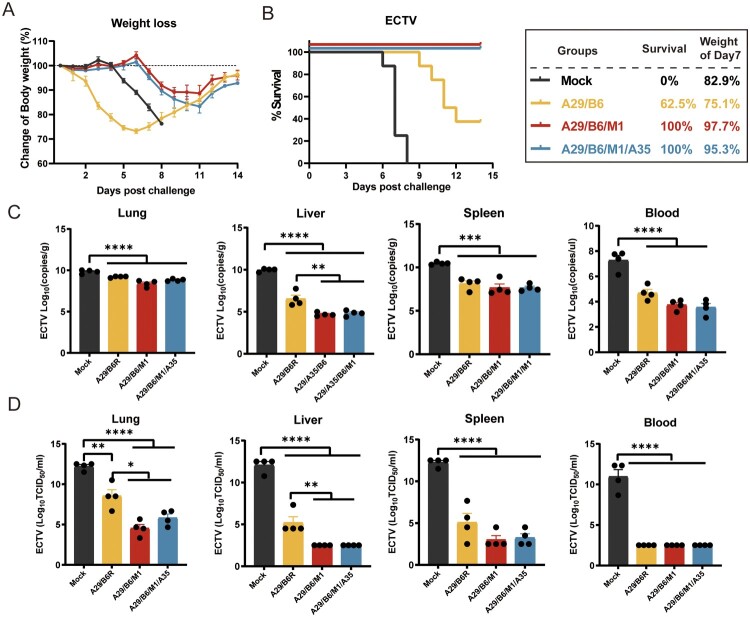

**Figure:**
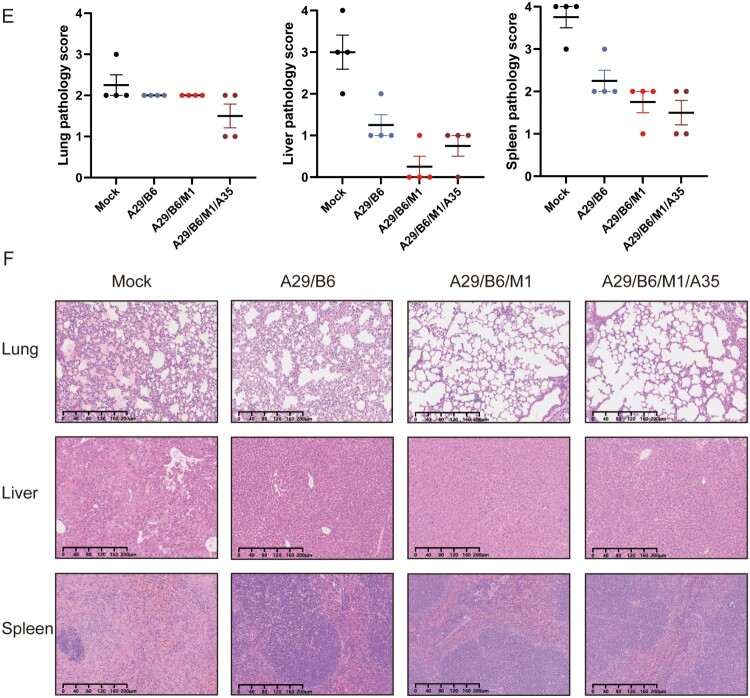

Pathological changes in the lungs, liver, and spleen of ECTV-infected mice were assessed at 7 DPI (Figure 3(E,F)). After intranasal administration of a high dose of ECTV, the lung, the liver and spleen of the mice in the control group showed obvious lesions (Figure 3(F)). Severe liver damage was observed in the unvaccinated mice, affecting almost all the liver parenchyma. Hepatocytes displayed considerable disarray, with widespread degeneration and necrosis; some showed indistinct borders, ruptured membranes, and cytoplasmic leakage, nuclear pyknosis, fragmentation, and dissolution were evident. In the spleen, there was substantial red blood cell accumulation beneath the capsule, a reduction in white pulp, and diffuse lymphocyte necrosis. Additionally, unvaccinated mice developed moderately severe interstitial pneumonia, with lung tissue necrosis, infiltration of inflammatory cells, and hemorrhage in the pulmonary interstitium. In contrast, mice immunized with the multicomponent MPXV protein vaccines (A29/B6/M1, A29/B6/M1/A35) exhibited only mild pathological changes in the lungs, liver, and spleen.

In summary, the multicomponent MPXV protein vaccines, A29/B6/M1 and A29/B6/M1/A35, provided complete cross-protection against high-lethal doses of ECTV.

### MPXV recombinant protein vaccines induced high IgG antibody titers, and some induced robust neutralizing activity against MPXV, VACV-VTT, and ECTV

**Figure 4.**
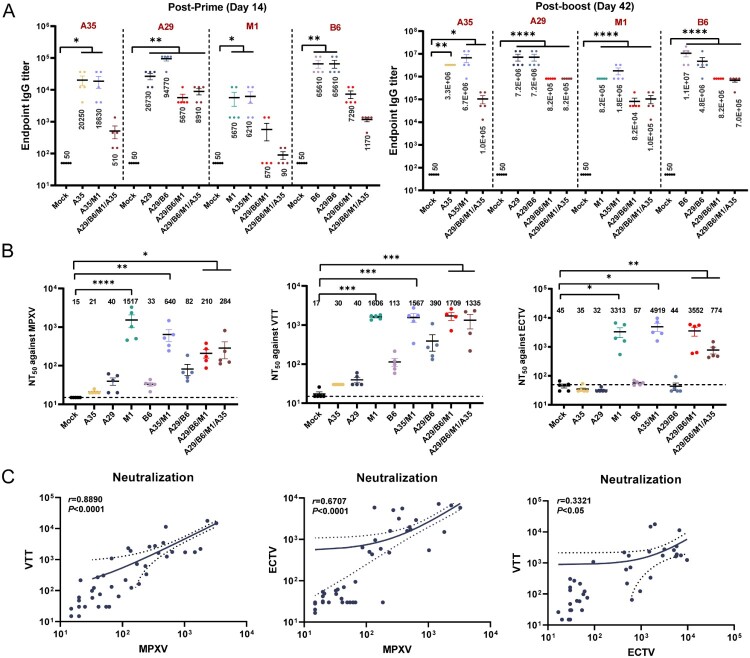
Humoral immune response in mice after inoculation with recombinant MPXV subunit protein vaccines. (A) Endpoint IgG titers as determined using ELISA in serum samples collected 2 weeks after each immunization (Days 14 and 42), targeting MPXV antigens A29L, B6R, M1R, and A35R. (B) Neutralizing antibody titers against VTT, ECTV, and MPXV were induced post-immunization and assessed at Day 42 using PRNT for MPXV and firefly luciferase-based methods for VTT and ECTV (VTT-Luc and ECTV-RLuc, respectively). Results are reported as the mean ± SEM and were analyzed using t-test or one-way ANOVA with correction for multiple comparisons, with *p* < 0.05 indicating statistical significance. (C) Correlation between neutralizing titers for VTT, ECTV, and MPXV, analyzed using logarithmic transformations and regression models. Predictions are indicated with a 95% confidence interval.

Serum samples were collected from mice on Days 14 and 42 after the initial immunization to measure IgG antibodies (Figure 4(A)). Both the single-component and multicomponent protein MPXV vaccines were effective in inducing specific IgG antibodies against MPXV. Following the initial immunization, the binding antibody titers elicited by M1 and B6 in the A29/B6/M1 and A29/B6/M1/A35 groups were relatively low (Figure 4(A)). After administering a second dose of vaccine, the binding antibody levels increased significantly in all groups, although the tri-component and quadruple-component protein vaccines induced slightly lower binding antibody levels than those induced by single protein immunization.

We then assessed the neutralizing antibody levels elicited by the MPXV recombinant protein vaccines using the PRNT and luciferase-based neutralization tests. Significant inhibition against MPXV was observed in the M1, A35/M1, A29/B6/M1, and A29/B6/M1/A35 groups (Figure 4(B)). The geometric mean of the neutralizing antibody titer in the M1 group was 1517, with a maximum titer of 3268. Given the high degree of sequence conservation between MPXV and VACV-VTT, we aimed to determine whether the MPXV protein vaccine-induced antibodies that were cross-reactive with VACV-VTT and ECTV. Consistent with the neutralization results against MPXV, the M1, A35/M1, A29/B6/M1, and A29/B6/M1/A35 groups exhibited high levels of cross-reactive antibodies with inhibitory activity against VACV-VTT and ECTV. Correlation analysis revealed a strong correlation between MPXV and VACV-VTT, a moderate correlation between MPXV and ECTV, and a weak correlation between VACV-VTT and ECTV (Figure 4(C)).

### MPXV recombinant protein subunit vaccines induced an MPXV-specific cellular immune response

**Figure 5.**
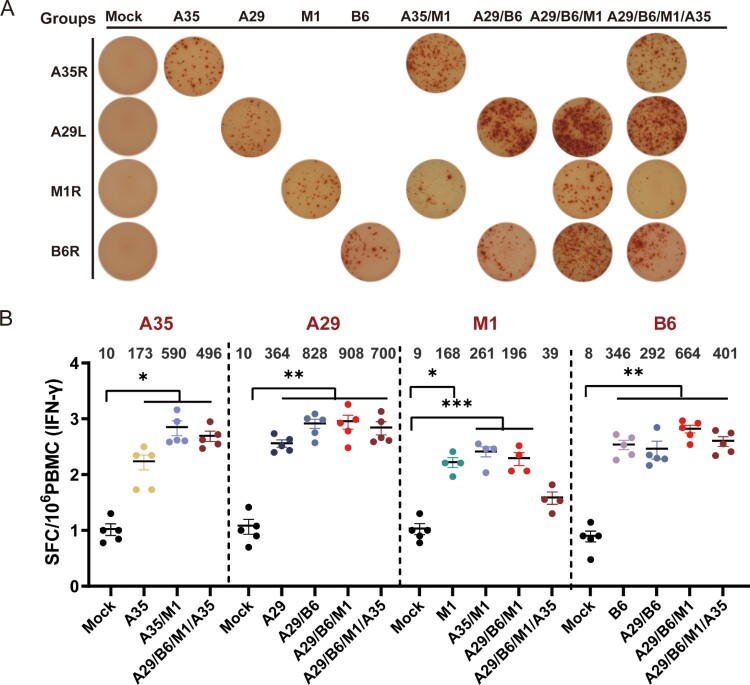
Detection of cellular immune response in mice after inoculation with recombinant MPXV subunit protein vaccine using IFN-γ ELISpot Cellular responses evaluated using IFN-γ ELISpot in spleens dissected 2 weeks after the second dose of vaccine. Spot-forming cells were detected following re-stimulation with the recombinant proteins A29L, B6R, M1R, and A35R. (A) Representative ELISpot images of spleen and inguinal lymph nodes (5 × 10^5^ cells). (B,C) Quantification of IFN-γ-producing cells in spleen (B) and lymph nodes (C). Data are represented as the mean ± SEM and were analyzed using t-tests or one-way ANOVA with correction for multiple comparisons, with *p* < 0.05 indicating statistical significance.

Given the critical role of T-cell immunity in defending against viral infections, the secretion of MPXV-specific interferon-gamma (IFN-γ) lymphocytes in the spleen was detected using enzyme-linked immunospot (ELISpot) on Day 42 after primary immunization. Both the single-component and multicomponent MPXV protein vaccines induced the production of MPXV-specific IFN-γ cells. The A35R protein did not enhance the cellular immune response to the tri-component (A29/B6/M1) protein group (Figure 5(A,B)). The addition of multicomponent proteins did not affect the secretion of MPXV IFN-γ cells stimulated by different proteins, other than in the A29/A35/B6/M1 group, which produced few IFN cells stimulated by M1. These results suggest that A35, A29, M1, and B6 in the protein vaccine can stimulate an effective T-cell immune response.

### Neutralizing activity and A35-/A29-/B6-specific cellular immunity appear to provide complementary mechanisms of protection

**Figure 6.**
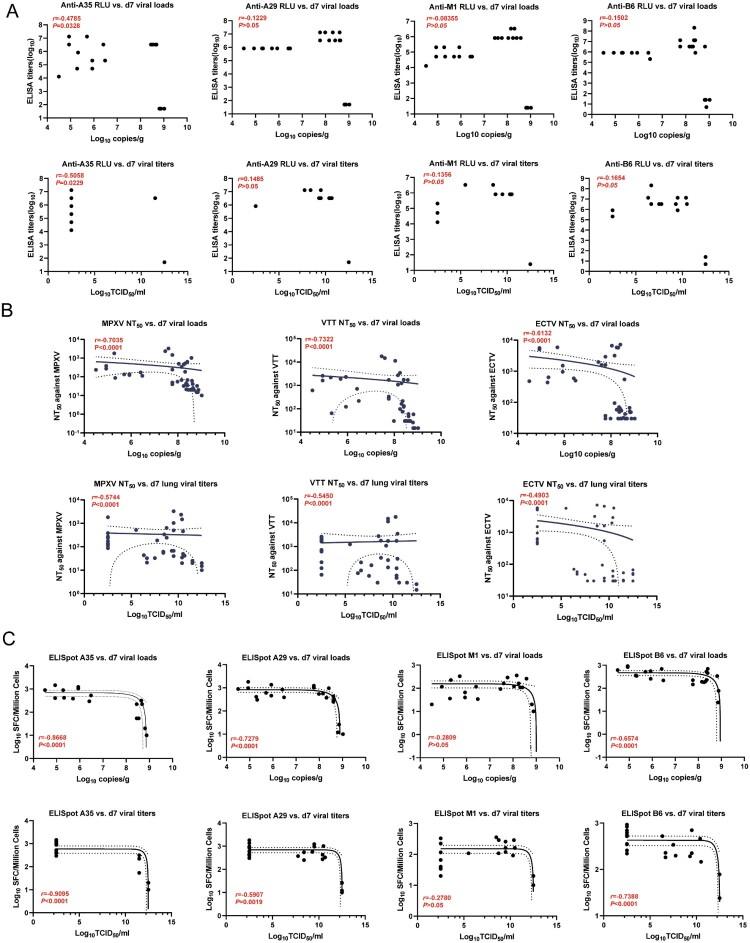
Vaccine immunogenicity negatively correlates with viral titer and viral load (A,B) Correlations between binding antibody responses to A35R, A29L, M1R, B6R, and viral titer and viral load at 7 days post infection. (B) Correlations between MPXV, VACV-VTT, ECTV neutralizing antibody responses and viral titer and viral loads (B) Correlations between cellular immune responses (IFN-γ) and viral titer and viral loads. Correlation analysis was performed using Spearman coefficients. *P* < 0.05 represents statistical significance. Estimates are shown with a 95% confidence interval.

To understand the respective contributions of neutralizing antibodies, antibody binding, and cellular immunity to the inhibition of MPXV replication, we tested for immune correlates of protection by comparing vaccine responses before the challenge with viral loads on Day 7 after the VACV-WR challenge. The binding antibody responses to A35 were negatively correlated with the viral load and virus titer at 7 DPI. In contrast, binding antibodies for A29, M1, and B6 showed no correlation with viral load and virus titer at 7 DPI (Figure 6(A)). Moreover, the MPXV virus-specific neutralizing antibody response negatively correlated with the viral load and virus titer at 7 DPI (Figure 6(B)). Similarly, the cross-neutralizing antibody responses specific to VACV-VTT and ECTV were negatively correlated with viral load and virus titer. Further correlation analysis of the specific cellular immunity generated by the MPXV protein vaccine indicated that the cellular immune responses induced by A35, A29, and B6 were negatively correlated with viral replication capacity, whereas M1 did not show any correlation (Figure 6(C)).

## Discussion

In this study, MPXV antigen proteins A29L, B6R, M1R, and A35R were modified, expressed, and purified using eukaryotic expression systems. BALB/c mice were inoculated with subunit vaccines derived from the eukaryotically expressed individual proteins (A35R, A29L, B6R, and M1R) or multicomponent combinations thereof with an Al(OH)_3_ + CpG ODN adjuvant. Each protein elicited a high-level protein-specific IgG response and demonstrated neutralizing activity against MPXV, VACV-VTT, and ECTV. Immunization with multicomponent protein vaccines (A29/B6/M1 and A29/B6/M1/A35) provided complete protection against high-lethal doses of VACV-WR and ECTV, administered intranasally. Additionally, we analyzed the roles of humoral and cellular immunity induced by different proteins in vaccine-mediated protection. Protective efficacy correlation analysis suggested that neutralizing activity and A35-/A29-/B6-specific cellular immunity function synergistically to offer complementary immune defense mechanisms, potentially providing first- and second-line protection against MPXV and other related orthopoxviruses. The multicomponent protein vaccine A29/B6/M1 showed the best overall protective efficacy against lethal challenge with VACV-VTT or ECTV in mice. This study provides insights on immunogen optimization and a potential strategy for developing novel vaccines against MPXV and other orthopoxviruses.

Recombinant protein subunits are considerably safer than live-virus vaccines and can provide satisfactory protection when used with adjuvants [27,42,43]. Previous studies have examined the efficacy of protein subunit vaccines against orthopoxviruses linked to various adjuvants and antigens [27,42,48–53]. Subunit protein vaccines containing A29L, B6R, M1R, and A35R, combined with adjuvants such as Al(OH)_3_ or CpG 7909 for intramuscular administration [53], or QS-21 for subcutaneous administration, trigger high levels of antigen-specific antibodies and cellular immune responses [42]. Additionally, Al(OH)_3_ and CpG ODN serve as adjuvants for MPXV/VACV protein vaccines, with CpG enhancing cell-mediated immunity and Al(OH)_3_ promoting humoral immunity [21,27,53]. In this study, we used Al(OH)_3_ + CpG ODN adjuvant with MPXV protein mixtures (A29/B6/M1/A35) for intramuscular administration in mice. Additionally, we assessed the immunogenicity and protective efficacy of other novel adjuvants (AddaS03 and WGa01) combined with MPXV proteins, but these adjuvants were less efficacious than the Al(OH)_3_ + CpG ODN adjuvant combination (Figures S2 and S3). Rational design of recombinant vaccines requires optimizing the immunogenicity of the selected Orthopoxvirus proteins. To better understand the contribution of each MPXV antigen-specific response to *in vivo* protection and to facilitate the scientific design of MPXV vaccines, we immunized mice with four selected immunogenic proteins (A35R, A29L, B6R, M1R), either individually or in multicomponent combinations, combining the proteins with Al(OH)_3_ + CpG ODN adjuvants.

The three multicomponent protein vaccines (A29/B6, A29/B6/M1, and A29/B6/M1/A35) provided complete protection against a challenge with a high-lethal dose of VACV-WR, whereas the other vaccines tested (individual proteins and the A35/M1 combination) provided only partial protection. A29/B6/M1 and A29/B6/M1/A35 showed superior protection to that of A29/B6. To evaluate the cross-protective efficacy of MPXV protein vaccines against orthopoxviruses, we tested ECTV IN BALB/c mouse challenge models [54]. This model closely mimics key clinical features of human Orthopoxvirus infections, such as smallpox, including acute systemic disease at low infectious doses, early absence of pulmonary involvement, viremia, and fever [55]. Additionally, because BALB/c mice are highly susceptible to ECTV infection, an ECTV challenge amplifies differences in the efficacy of the different vaccines. Following a lethal ECTV challenge, mice immunized with A29/B6/M1 or A29/B6/M1/A35 experienced mild, transient weight loss, lower viral load, reduced viral copy number, and less pathological damage in various tissues following a lethal ECTV challenge compared with the control group. In contrast, A29/B6 failed to confer complete protection. In the control and A29/B6 groups, ECTV replicated in local lymph nodes, leading to primary viremia and subsequent dissemination to the liver and spleen [56]. This lower protective efficacy may be attributable to the highly stringent challenge conditions used in this model. These findings suggest that the M1 component plays a crucial role in preventing IMV entry into host cells and may contribute to the neutralization of both the initial inoculum and progeny viruses [29].

To explore the immune mechanisms underlying the protection conferred by MPXV protein vaccines, we performed a correlation analysis between each antibody characteristic and viral replication post-challenge. Both the single-component and multicomponent MPXV vaccines induced specific binding antibodies against MPXV after primary immunization, with levels increasing after administering the second dose of the vaccine. Correlation analysis indicated that after immunization with A35, A35-binding antibody levels were negatively correlated with virus protection. This was not observed with the A29, B6, or M1 vaccines. A35 provided poor protection, with only a 12.5% survival rate against the lethal VACV-WR challenge. This suggests that binding antibodies may not be a reliable immune indicator of the protective efficacy of MPXV protein vaccines.

The neutralizing antibody to vaccination is a necessary and sufficient component of smallpox vaccine-mediated protective immunity [57,58]. In our study, we observed a strong negative correlation between MPXV, VACV-neutralizing antibodies, and protective efficacy. However, only mice who received the M1 antigen (including M1, A35/M1, A29/B6/M1, and A29/B6/M1/A35) were capable of generating neutralizing antibodies specific to MPXV, and cross-neutralizing antibodies against VACV-WR and ECTV, highlighting the dominance of M1 at promoting the virus neutralizing activity of the vaccine [27,41,59]. The A29/B6/M1 vaccine provided better protection than that of the A29/B6 vaccine, further highlighting the importance of M1 as an immunogen in MPXV vaccine design. However, we observed that, although M1 is crucial for generating neutralizing antibodies (NT_50_ = 1517), the protection rate of M1 alone was only 57.1%. In contrast, the B6 vaccine, despite generating low neutralizing antibody levels, showed a protection rate of 62.5%. This suggests that individual proteins may not offer complete protection to mice against viral infection, as antibodies may only act on a single stage of the viral life cycle (EEV or IMV), consistent with previous research validating MPXV/VACV single antigens [22,41,60].

Antigen-specific T-cell responses play a critical role in various vaccines in early viral clearance, control of disease severity, limiting viral transmission, and are an essential component of vaccine efficacy [61]. We detected substantial MPXV antigen-specific IFN-γ cytokine production in mouse spleen cells following the second dose of vaccine, particularly in response to A29- and B6-stimulated IFN-γ production. The IFN-γ cytokines induced by A35, B6, and A29 antigens were negatively correlated with viral replication, suggesting that cellular immunity is an important indicator of protection against orthopoxviruses. The A29/B6 protein immune group provided protection against a lethal VACV-WR challenge despite A29 and B6 not inducing significant neutralizing antibody titers. Xu et al. [62] demonstrated that following acute infection, CD4^+^ T-cell-dependent antibody responses are indispensable for the clearance of VACV, while in the absence of CD4^+^ T-cells and antibodies, CD8^+^ T-cells can provide mice with significant protection from disease. Additionally, a coordinated and robust response from both CD4^+^ and CD8^+^ T-cells is associated with milder disease [63]. Furthermore, recent studies on MPXV mRNA vaccines have highlighted the role of antibody-mediated Fc effector functions – such as antibody-dependent cellular cytotoxicity, antibody-dependent cellular phagocytosis, antibody-dependent complement deposition, and antibody-dependent neutrophil phagocytosis – in controlling disease progression [41,64]. Collectively, these findings suggest that humoral immunity and cellular immunity function in a coordinated manner to provide orthogonal mechanisms of immune control, potentially offering first-line and second-line immune protection against MPXV and related orthopoxviruses. However, further exploration of the intricate mechanisms underlying the immune response to MPXV and other orthopoxviruses is required.

Our study showed that the A29/B6/M1 multicomponent protein vaccine provided comparable or superior protection to that of the A29/B6/M1/A35 group. Two intramuscular doses conferred complete protection against a challenge with VACV-WR or ECTV in mice, significantly reducing viral infection in mouse tissues. Our study demonstrates that a combination of at least these three antigens is required for MPXV vaccine optimization and antigen-saving purposes. In this study, A35R did not appear to play a direct role in inducing an immune response to MPXV protein vaccines. Even when each protein was administered at an identical dose (10 μg per protein), the A29/B6/M1 vaccine demonstrated higher protective efficacy than that of the A29/B6/M1/A35 vaccine. This was reflected in the degree of weight loss and viral titers in lung tissue following VACV-WR and ECTV infection. We hypothesize that receptor competition among proteins may interfere with their interaction, leading to diminished efficacy [27,38]. In addition, a study of seven VACV A33-specific monoclonal antibodies [65] found that, of the five antibodies that neutralized VACV in the presence of complement, only A27D7, which recognizes a dimeric epitope, was a potent cross-neutralizing antibody. The stalk region is dispensable for the dimerization of A35^Ecto^ in vitro but is required for its immunogenicity. Another study designed two copies of the soluble A35 antigen in the immunogen to mimic the natural dimeric structure of A35R and enhance A35R-specific immunogenicity [66]. This implies that the monomeric form of A35R in our vaccine formulation may not closely mimic its natural conformation, thus limiting its ability to trigger a robust protective immune response. Further studies of the A35 protein, including potential modifications to enhance its immunogenicity, are needed to investigate the immunogenicity of the A35 protein in MPXV vaccines. Additionally, evaluating the protective efficacy of lower doses of multicomponent protein vaccines and their effectiveness at preventing MPXV infection is essential.

In summary, our study evaluated the immunogenicity and protective efficacy of the MPXV A35R, A29L, M1R, and B6R proteins, either individually or in multicomponent formulations. We assessed correlations between the antigenic immune response of MPXV proteins with their protective effects. The results suggest that humoral and cellular immune functions may play complementary roles in immune defense against MPXV and other orthopoxviruses. Additionally, we developed the A29/B6/M1 and A29/B6/M1/A35 protein vaccines, which elicited potent neutralizing antibodies against MPXV, VACV, and ECTV, provided comprehensive protection against lethal doses of VACV-WR and ECTV, and significantly reduced viral loads in mouse tissues. Further MPXV challenge studies in CAST/EiJ mice and nonhuman primates are necessary to evaluate antigen-specific immune responses and elucidate the protective mechanisms of each vaccine candidate before advancing to clinical trials.

People living with HIV have an increased risk of severe MPXV infection [67]. These protein subunit vaccines, which provide improved control and accessibility, have the potential to serve as next-generation vaccines against MPXV and other orthopoxviruses. Furthermore, the complete protection conferred by the A29/B6/M1 and A29/B6/M1/A35 protein vaccines against ECTV, a more distantly related lineage, suggests that these protein subunit vaccines could form the foundation for pan-Orthopoxvirus vaccines, including those for smallpox and disease “X” caused by VACV.

## Supplementary Material

supplements.pdf
