# Supplementary material for: Rational mpox vaccine design: immunogenicity and protective effect of individual and multicomponent proteins in mice
Source: Emerg Microbes Infect. 2025 Mar 19;14(1):2482702. doi: 10.1080/22221751.2025.2482702 (PMC11951338; doi:10.1080/22221751.2025.2482702)
Supplement: supplements.pdf [file TEMI_A_2482702_SM1081.pdf]

Supplementary Materials

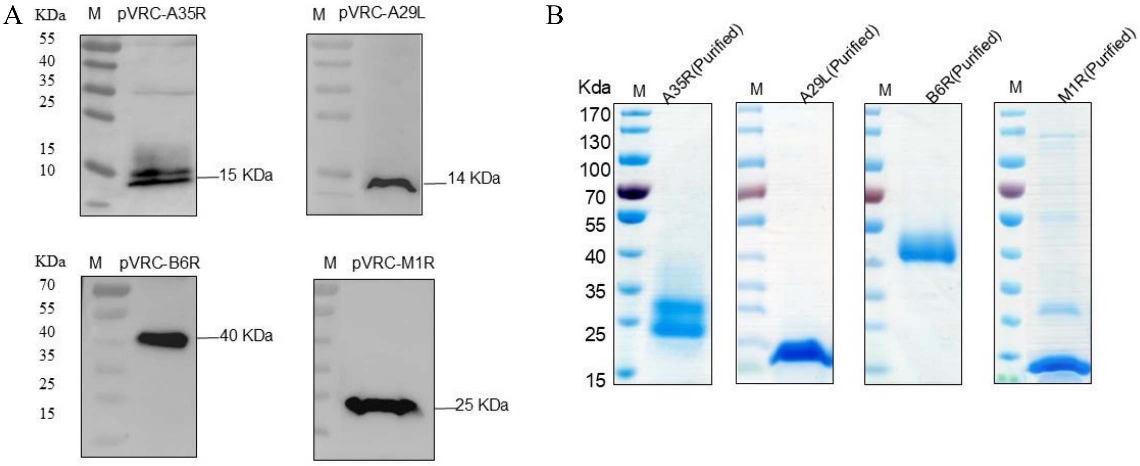

Figure S1. Verification and purification of MPXV A35R, A29L, B6R, M1R protein (A) The results of Western blotting after MPXV A35R, A29L, B6R, M1R plasmid transfection into 293T cells. (B) The results of Coomassie Brilliant Blue staining after protein purification.

A

Groups	Vaccines	Proteins
1	Mock	50μl PBS
2	A29+B6+Al(OH)$_3$	10μg*2
3	A29+B6+AddaS03	10μg*2
4	M1+A35+Al(OH)$_3$	10μg*2
5	M1+A35+AddaS03	10μg*2

B

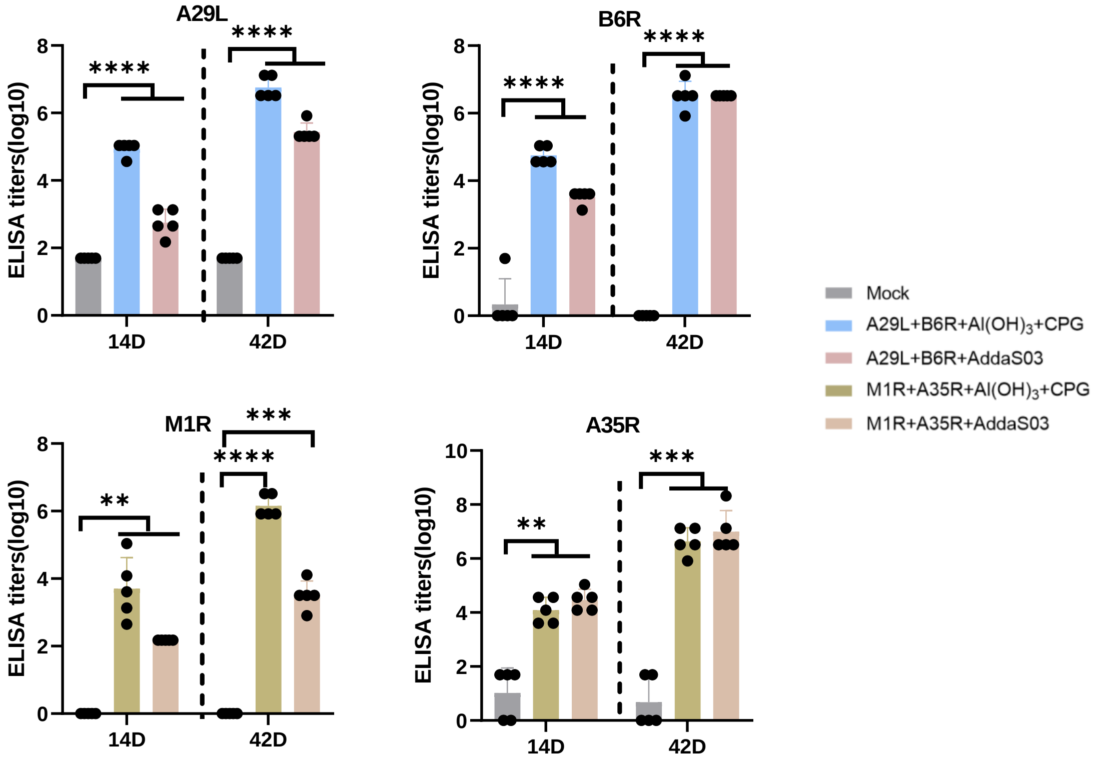

C

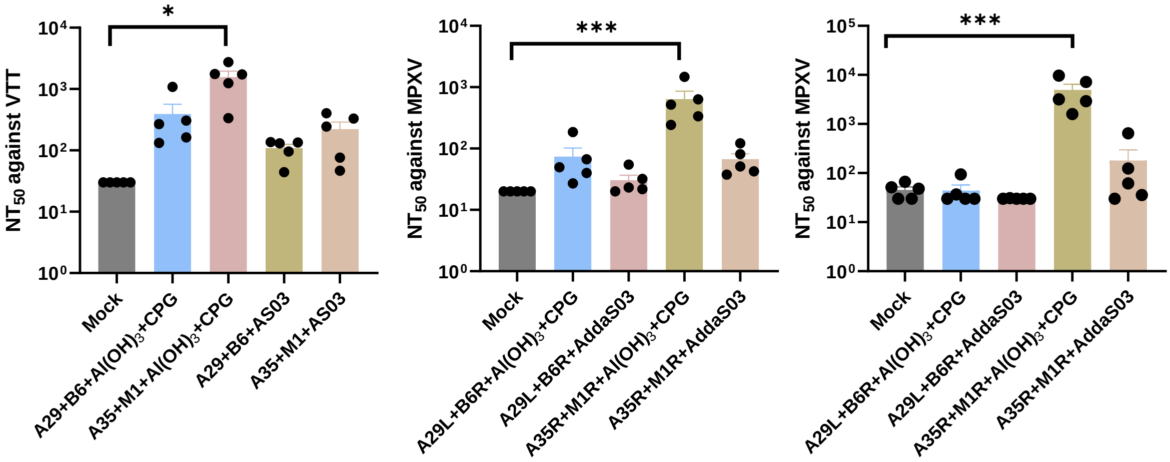

D

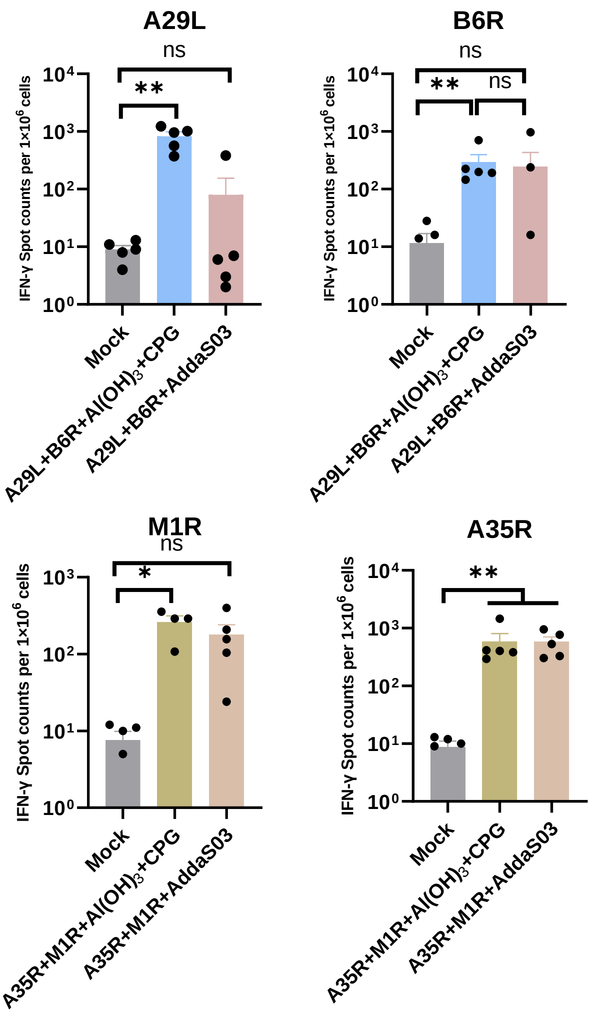

A29L
B6R
M1R
A35R
ns
**
*
IFN-γ Spot counts per 1×10^6 cells
Mock
A29L+B6R+Al(OH)3+CPG
A29L+B6R+AddaS03
A35R+M1R+Al(OH)3+CPG
A35R+M1R+AddaS03

E

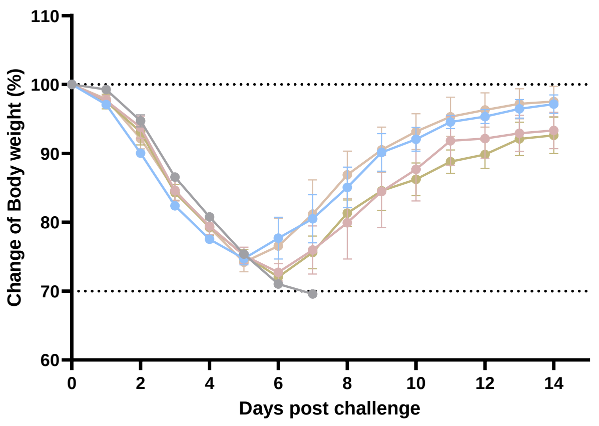

Change of Body weight (%)
Days post challenge

F

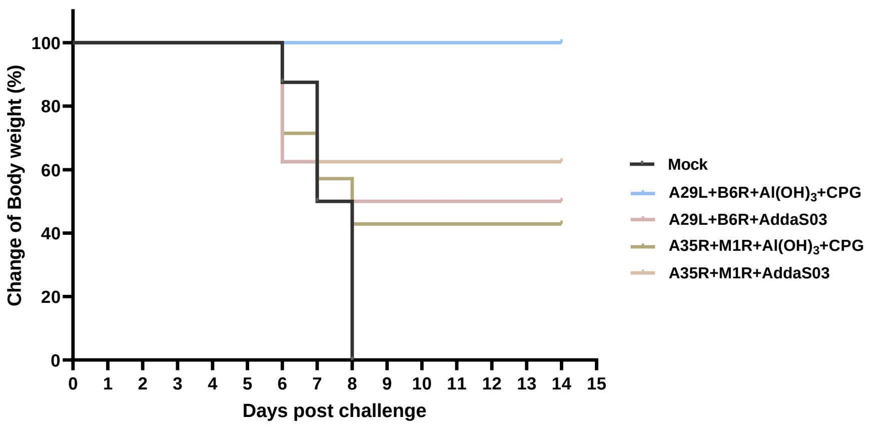

Change of Body weight (%)
Days post challenge
Mock
A29L+B6R+Al(OH)3+CPG
A29L+B6R+AddaS03
A35R+M1R+Al(OH)3+CPG
A35R+M1R+AddaS03

G

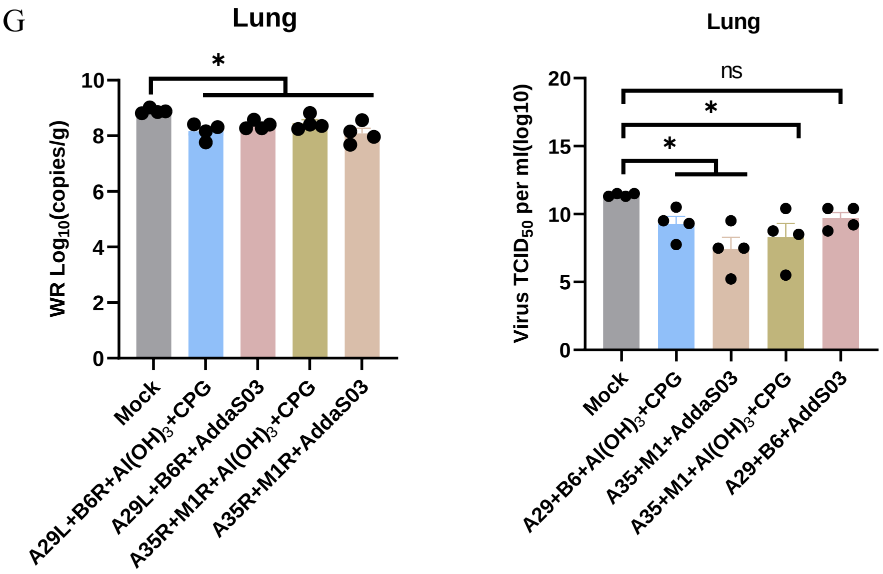

Figure S2. Cross - protection of tetravalent MPXV subunit protein vaccines with $Al(OH)_3$ + CpG ODN or AddaS03 adjuvants against lethal ECTV challenge in mice (A) Grouping of Mice Immunized with Proteins vaccine. (B) Endpoint IgG titres as determined using ELISA in serum samples collected 2 weeks after each immunisation (Days 14 and 42), targeting MPXV antigens A29L, B6R, M1R, and A35R. (C) Neutralising antibody titres against VTT, ECTV, and MPXV were induced post-immunisation and assessed at Day 42 via PRNT for MPXV and firefly luciferase-based methods for VTT and ECTV (VTT-Luc, ECTV-RLuc). (D) Cellular responses evaluated using IFN-γ ELISpot in spleens dissected 2 weeks after the second dose of vaccine. Spot-forming cells were detected following re-stimulation with the recombinant proteins A29L, B6R, M1R, and A35R. (E) Changes in body weight and (F)survival after intranasal challenge with 15 LD_{50} of VACV-WR on Day 7 after administering the second dose of vaccine evaluated in mice vaccinated with subunit vaccines. (G) Virus copies numbers and virus titres in the lung 7 days post-infection.

A

Groups	vaccines	Protein
1	Mock	50μlPBS
2	A29+A35+B6+M1+Al(OH)$_3$+CpG	10μg*4
3	A29+A35+B6+M1+WGa01	10μg*4

B

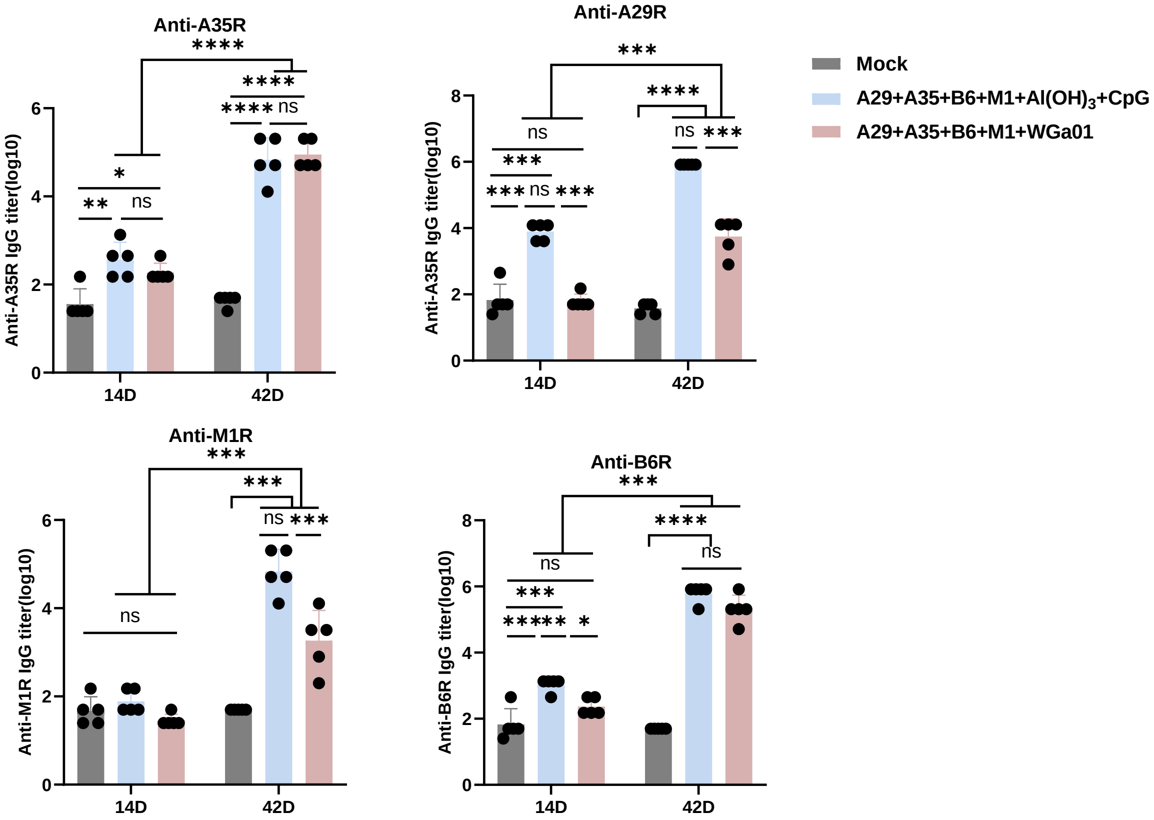

C

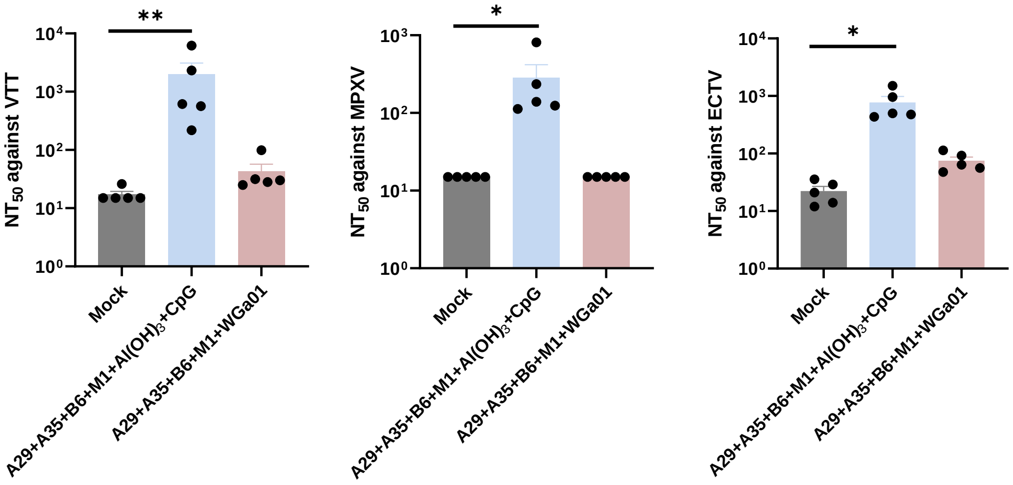

D

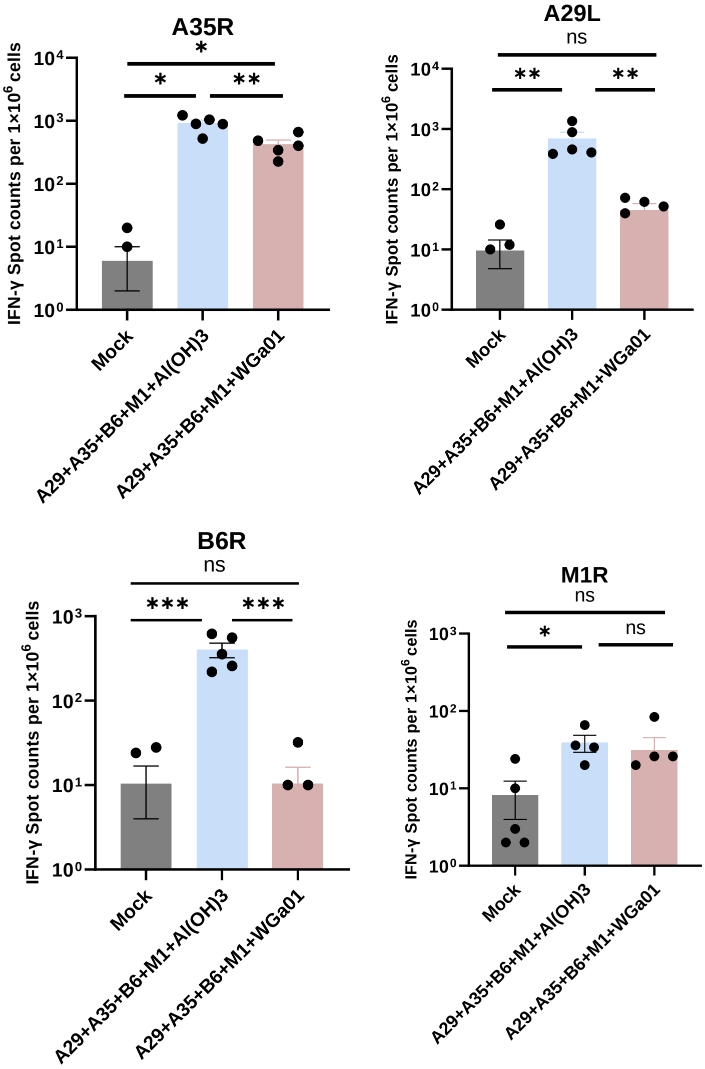

A35R
*
*
**
IFN-γ Spot counts per 1×10^6 cells
Mock
A29+A35+B6+M1+Al(OH)3
A29+A35+B6+M1+WGa01
A29L
ns
**
**
IFN-γ Spot counts per 1×10^6 cells
Mock
A29+A35+B6+M1+Al(OH)3
A29+A35+B6+M1+WGa01
B6R
ns
***
***
IFN-γ Spot counts per 1×10^6 cells
Mock
A29+A35+B6+M1+Al(OH)3
A29+A35+B6+M1+WGa01
M1R
ns
*
ns
IFN-γ Spot counts per 1×10^6 cells
Mock
A29+A35+B6+M1+Al(OH)3
A29+A35+B6+M1+WGa01

E

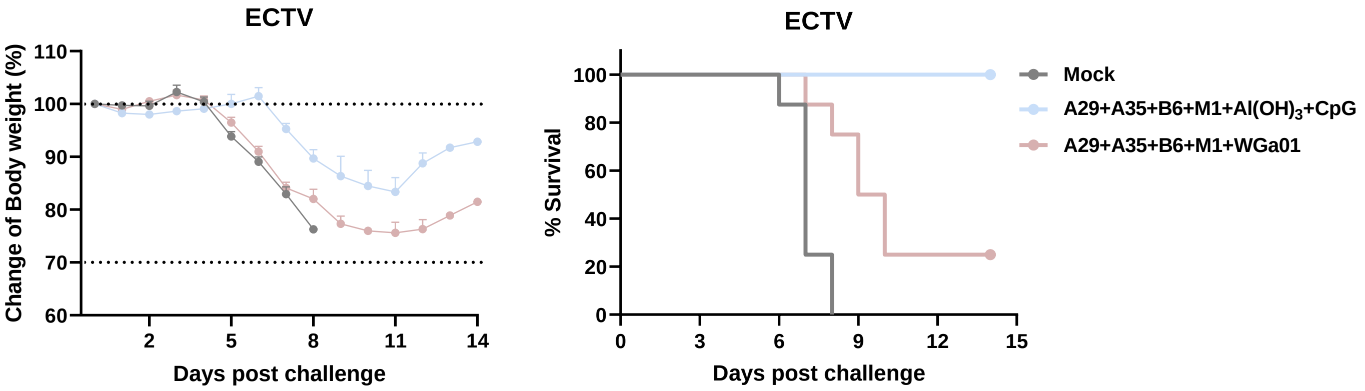

ECTV
Change of Body weight (%)
Days post challenge
ECTV
% Survival
Days post challenge
Mock
A29+A35+B6+M1+Al(OH)3+CpG
A29+A35+B6+M1+WGa01

F

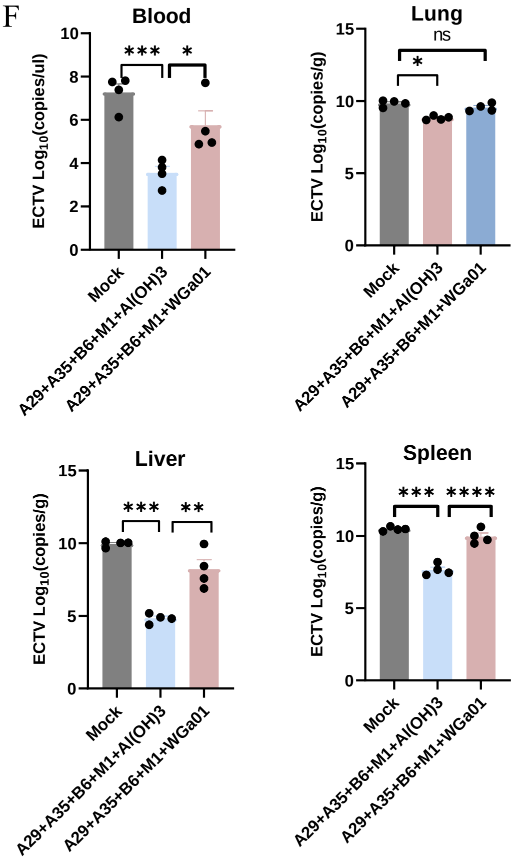
Blood
ECTV Log10(copies/ul)
*** *
Lung
ns
*
ECTV Log10(copies/g)
Liver
*** **
Spleen
*** ****
Mock
A29+A35+B6+M1+Al(OH)3
A29+A35+B6+M1+WGa01

G

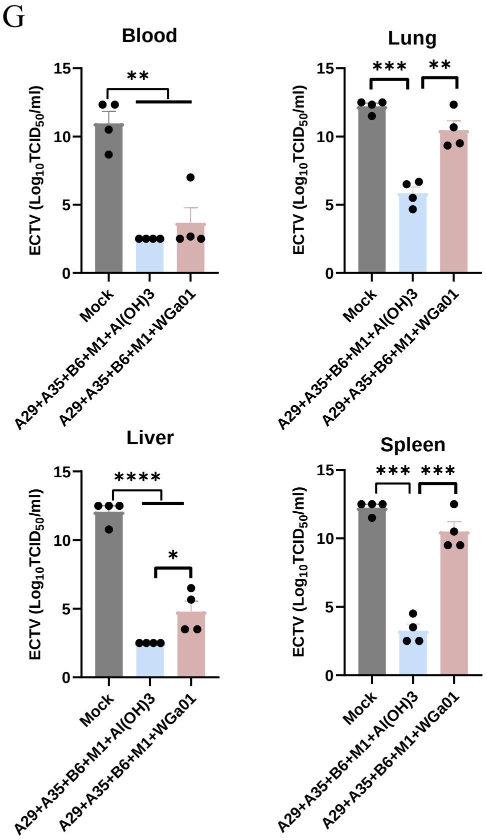
Blood
**
ECTV (Log10TCID50/ml)
Lung
*** **
Liver
****
*
Spleen
*** ***
Mock
A29+A35+B6+M1+Al(OH)3
A29+A35+B6+M1+WGa01

H

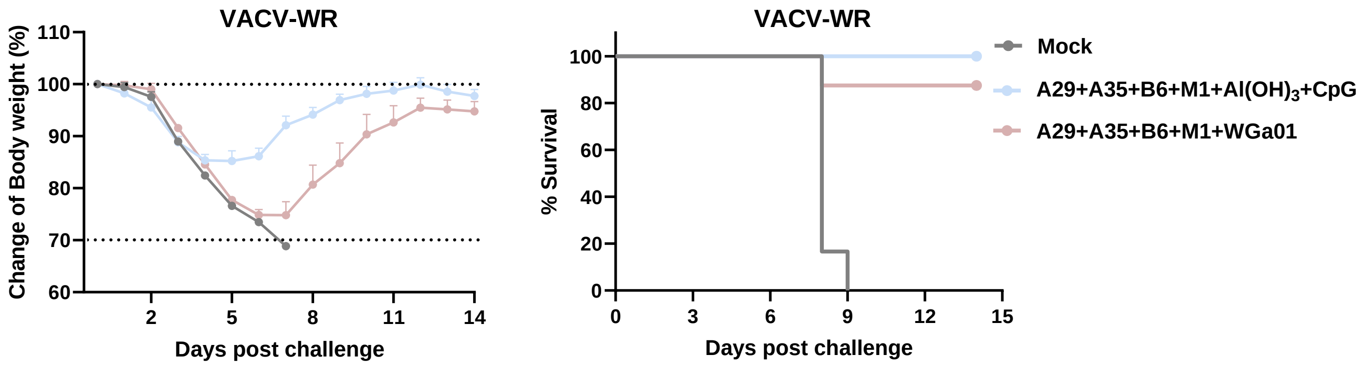
VACV-WR
Change of Body weight (%)
Days post challenge
VACV-WR
% Survival
Days post challenge
Mock
A29+A35+B6+M1+Al(OH)3+CpG
A29+A35+B6+M1+WGa01

I

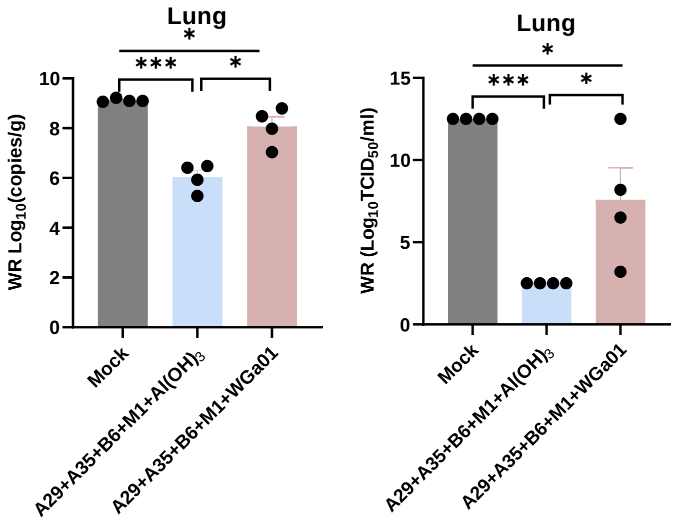

Figure S3. Cross - protection of divalent MPXV subunit protein vaccines with $Al(OH)_3$ + CpG ODN or WGa01 adjuvants against lethal ECTV challenge in mice. (A) Grouping of Mice Immunized with Proteins vaccine. (B) Endpoint IgG titres as determined using ELISA in serum samples collected 2 weeks after each immunisation (Days 14 and 42), targeting MPXV antigens A29L, B6R, M1R, and A35R. (C) Neutralising antibody titres against VTT, ECTV, and MPXV were induced post-immunisation and assessed at Day 42 via PRNT for MPXV and luciferase-based methods for VTT and ECTV (VTT-Luc, ECTV-RLuc). (D) Cellular responses evaluated using IFN-γ ELISpot in spleens dissected 2 weeks after the second dose of vaccine. Spot-forming cells were detected following re-stimulation with the recombinant proteins A29L, B6R, M1R, and A35R. (E) Changes in body weight and survival after intranasal challenge with 15 LD_{50} of ECTV on Day 7 after administering the second dose of vaccine evaluated in mice vaccinated with subunit vaccines. (F) Virus copies numbers and (G) virus titres in the lung, liver, spleen, and blood 7 days post-infection. (H) Changes in body weight and survival after intranasal challenge with 15 LD_{50} of VACV-WR on Day 7 after administering the second dose of vaccine evaluated in mice vaccinated with subunit vaccines. (I) Virus copies numbers and virus titres in the lung 7 days post-infection. Data are expressed as the mean±SEM and were analysed using one-way ANOVA, with $P<0.05$ indicating statistical significance.

Table S1. MPXV target protein amino acid sequence consensus and identity with VACV and VARV orthologs

MPXV protein	Amino acid sequence length	VACV protein	VACV – identity consensus sequence length (identity [%])	VARV protein	VARV – identity consensus sequence length (identity [%])	molecular sizes	Function and characteristics	Protein ID (NCBI)	Ref.
A35	181	A33	172/180 (96%)	A36	166/180 (92%)	23-28kDa	EEV envelope glycoprotein; Formation of actin-containing microvilli and cell-to-cell spread of virion; C-type lectin-like domain	OPG161	[32]
A29	111	A27	104/110 (95%)	A30	103/110(94%)	14kDa	IMV surface fusion protein; Binds to cell surface heparan; Neutralizing antibody target	OPG154	[28]
B6	317	B5	306/317 (97%)	B7	294/316 (93%)	42kDa	EEV type-1 membrane glycoprotein; Required for efficient cell spread; target of protective antibody; Complement control	OPG190	[30,31]
M1	250	L1	246/250 (98%)	M1	248/250 (99%)	23-29kDa	Myristylated IMV surface membrane protein; Virus entry into a host; Neutralizing antibody target	OPG095	[29]
